# Reduced insulin-like signaling restores motor and chemosensory neuron function in *C. elegans* expressing TDP-43, an ALS-associated protein

**DOI:** 10.17912/micropub.biology.001788

**Published:** 2025-09-16

**Authors:** Vincent Lotesto, Cindy Voisine

**Affiliations:** 1 Department of Biology, Northeastern Illinois University, Chicago, IL, USA

## Abstract

TAR DNA-binding Protein 43 (TDP-43) is linked to the pathology of neurodegenerative diseases. We used the roundworm *Caenorhabditis elegans* to examine the neurotoxic impact of the pan-neuronal expression of wild-type human TDP-43 (hTDP-43) fused to a yellow fluorescent protein. Using the *daf-2 ( e1370 )* mutant allele, we sought to determine whether activating cellular stress responses in the insulin-like signaling (ILS) pathway could restore neuronal function in hTDP-43 expressing *C. elegans* . Using well characterized behavioral assays, our data show that manipulating the ILS pathway significantly improves functionality of motor and chemosensory neurons in animals expressing hTDP-43.

**Figure 1. Reduced insulin-like signaling improves motility and chemosensory function in hTDP-43 expressing animals:**
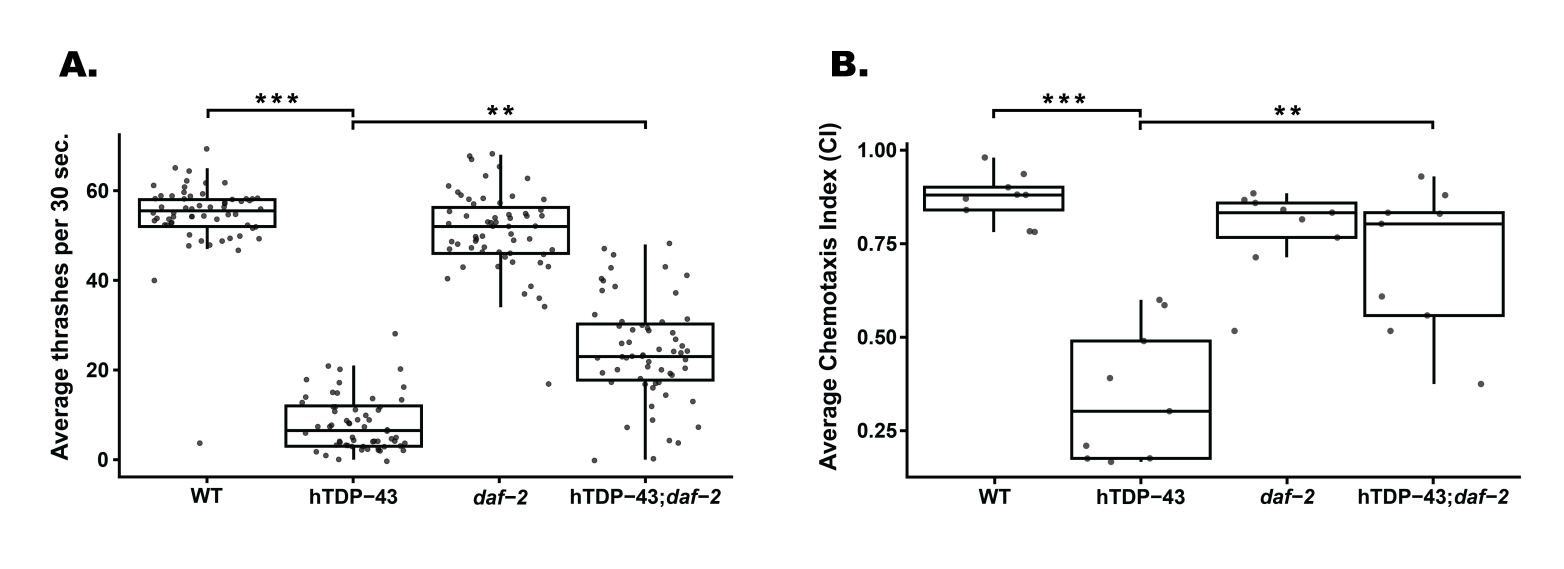
**(A)** The number of thrashes in 30 seconds were measured for wild-type (WT), hTDP-43, *daf-2 ( e1370 )* , and hTDP-43; *daf-2 ( e1370 )* animals. hTDP-43 showed a significant decrease in thrashes compared to WT animals (*** *p* <0.001). hTDP-43; *daf-2 ( e1370 )* showed a significant increase in thrashes compared to hTDP-43 expressing animals (** *p* <0.01). Data represents the average of 3 independent experiments, N=20 worms per replicate. Error bars represent SD. **(B)** hTDP-43 showed a significant decrease in Chemotaxis Index (CI) compared to WT animals (*** *p* <0.001). hTDP-43; *daf-2 ( e1370 )* showed a significant increase in CI value compared to hTDP-43 expressing animals (** *p* <0.01). Data represents the average of 3 independent experiments, with at least N=75 worms per replicate. Error bars represent SD.

## Description

*Caenorhabditis elegans* are a valuable *in vivo* model for studying age-related neurodegenerative diseases, such as amyotrophic lateral sclerosis (ALS). Ease of creating transgenic lines and using well established behavioral assays allow assessments of the neurotoxic impacts and physiological changes related to expression of the ALS-associated protein TDP-43 (Ash et al., 2010; Chen et al., 2024; Koopman et al., 2023; Van Pelt & Truttmann, 2020; Zhang et al., 2011, 2012). Previous findings have shown that pan-neuronal expression of wild-type human TDP-43 (hTDP-43) in *C. elegans* demonstrates neuronal deficits (Ash et al., 2010; Zhang et al., 2011, 2012). Proteotoxicity of hTDP-43 may be attenuated by the modulation of cellular stress pathways such as the evolutionarily conserved insulin-like signaling (ILS) pathway. Insulin-like signaling can be reduced using a mutation in the pathway's receptor, DAF-2 . ILS pathway modulation by the partial loss of function *daf-2 ( e1370 )* allele has been shown to enhance resistance to cellular stressors including proteotoxicity (Chen et al., 2024; Hsu et al., 2003; Kenyon et al., 1993; Lin et al., 2001; Prahlad & Morimoto, 2009; Tullet et al., 2008; Vaccaro et al., 2012). Thus, we aimed to examine if the mutant *daf-2 ( e1370 )* could alleviate the neuronal deficits observed in hTDP-43 in transgenic *C. elegans* .

Motility assays were used to determine if the *daf-2 ( e1370 )* mutant could restore motor neuron functionality in hTDP-43 expressing *C. elegans* . This behavior was assessed by counting the number of complete thrashes made by the animals within a time frame of 30 seconds. Wild-type (WT) animals demonstrated an average of 55 thrashes per 30 seconds compared to the hTDP-43 animals average of 8 thrashes per 30 seconds, exhibiting a significant decrease in motor neuron function compared to the WT control ( *p* <0.001) ( **Figure 1A** ). Animals carrying the *daf-2 ( e1370 )* mutant allele had an average of 51 thrashes per 30 seconds, exhibiting no significant decrease in thrashes compared to the WT control ( **Figure 1A** ). Importantly, the transgenic strain hTDP-43; *daf-2 ( e1370 )* was assessed to determine the impact of the *daf-2 ( e1370 )* mutant allele on motor neuron functionality. Compared to the hTDP-43 expressing animals, the hTDP-43 *; daf-2 ( e1370 )* strain exhibited a significant increase in thrashing rate with an average of 24 thrashes per 30 seconds ( *p* <0.01) ( **Figure 1A** ). This suggests that the *daf-2 ( e1370* ) mutation aids in attenuating the motor neuron deficits imposed by hTDP-43. Reduced transgene expression in the hTDP-43 *; daf-2 ( e1370 )* strain compared to the hTDP-43 transgenic line may explain the improvement in motility. However, visual inspection using fluorescence microscopy determined that transgene expression levels are similar in both strains with and without the mutant allele.

Following our assessment of motility, we postulated whether the *daf-2 ( e1370* ) mutation could be beneficial for attenuating the neurotoxicity in other neurons. For example, chemosensory deficits have been observed in hTDP-43 expressing *C. elegans* (Koopman et al., 2023). Chemotaxis assays were performed to assess the sensory behavior of our *C. elegans* strains. In the presence of the known chemical attractant isoamyl alcohol (Bargmann et al., 1993), a chemotaxis index (CI) value near positive 1 (+1) is expected. WT animals yielded an average CI value of 0.872 compared to the hTDP-43 animals average CI value of 0.32 ( **Figure 1B** ). These data demonstrate that hTDP-43 expressing animals have a significant reduction in chemosensory functionality in the presence of an attractant ( *p* <0.001). The *daf-2 ( e1370 )* mutant yielded a CI value of 0.782, exhibiting no significant difference compared to the WT control ( **Figure 1B** ). Animals expressing hTDP-43 and carrying the *daf-2 ( e1370* ) mutation exhibited a significant increase in sensory capabilities yielding a CI value of 0.71 ( *p* <0.01). ( **Figure 1B** ). This suggests that the *daf-2 ( e1370* ) mutation also aids in attenuating the chemosensory neuron deficits imposed by hTDP-43 expression.

Our experiments confirm previously documented neuronal deficits in hTDP-43 expressing *C. elegans* (Ash et al., 2010; Zhang et al., 2011, 2012) and attenuation of motility deficits through the *daf-2 ( e1370* ) mutation (Zhang et al., 2011). Additionally, our experiments show that the *daf-2 ( e1370* ) mutation attenuates sensory deficits in the presence of a chemical attractant. Taken together, our data demonstrate that the *daf-2 ( e1370* ) mutant background significantly restores functionality of both motor and chemosensory neurons in hTDP-43 expressing *C. elegans* .

## Methods

**Strains.** All hermaphroditic *C. elegans* strains were cultured at 20°C using *Escherichia coli* strain OP50 for a food source as previously described (Brenner, 1974; Lewis & Fleming, 1995). N2 strain was used as the wild-type control. Transgenic strains included AM1029 ( *rmIs348* [ *F25B3.3p::hTDP-43::yfp* ]), *daf-2 ( e1370 )* , and CMV0001 ( *rmIs348* [ *F25B3.3p::hTDP-43::yfp* ] *; daf-2 ( e1370 )* .

**Synchronization of *C. elegans* .** Gravid adults were harvested and prepared via a bleach (sodium hypochlorite) synchronization process using previously documented methods (Lewis & Fleming, 1995). Animals were transferred via a pasteur pipet into 15-ml centrifuge tubes and bleached using a 20% alkaline hypochlorite solution. Animals were washed twice using M9 buffer taking care to not disturb the resulting egg pellet. Egg pellets were resuspended in 1 ml M9 buffer and dispensed via pipette in respective 6 cm Petri dishes containing M9 buffer. Eggs were allowed to incubate at 20°C overnight to yield L1 stage animals. L1 animals were then replated on NGM plates containing OP50 and incubated once again at 20°C for 72 hrs in order to have day 1 gravid adult *C. elegans* for use in experimental assays.

**Motility assays.** Individual day 1 gravid adult *C. elegans* were placed in 15μL of M9 buffer solution and allowed an acclimation period of 30 seconds. Upon acclimation, the number of thrashes, or body bends, within a period of 30 seconds were scored. A thrash was defined as a complete lateral bend of the animal's body from one position to a secondary position. At least 60 animals were scored for each strain across three biological replicates.

**Chemosensory Assays.** Chemosensory assays were conducted using the quadrant plate method (Wicks & Rankin, 1995). A 60 mm petri dish was divided into quadrants and each quadrant had an application site; two of which were inoculated with a control of 1ul of ethanol and the other two were inoculated with a treatment of 1ul isoamyl alcohol (Sigma-Aldrich cat. #W205702), a known attractant. A paralytic agent of 1ul 1M sodium azide (Sigma-Aldrich cat. #S2002) was also administered to each of the four application sites. At least 75 day 1 adult *C. elegans* were placed within the center of the plate and were allotted 2 hrs to freely roam the plate. This was adapted from the 1hr timeframe as previously described (Bargmann et al., 1993) to account for the motor deficits of the animals. Upon time, paralyzed worms within an 18mm radius of each application site were scored. From these scores, a Chemotaxis Index (CI) value was determined using the following equation: *CI = (A-B)/(A+B)* where, *A* represents the number of animals at the odorant sites (application sites with isoamyl alcohol), *B* represents the number of animals at the control sites (application sites with ethanol). A positive CI value (CI>0), is interpreted as an attractive response to the odorant. A negative CI value (CI<0), is interpreted as an aversive response to the odorant.

**Statistical Analysis.** Statistical significance of differences for both assays were evaluated via One-way ANOVA followed by a Tukey's HSD test using R (v4.4.1). Data sets were expressed as means ± standard deviation (SD).

## Reagents

**Table:** 

**Strain**	**Genotype**	**Reference**
Bristol N2	Wild-type *C. elegans*	CGC
CB1370	*daf-2 ( e1370 )* III.	CGC
AM1029	*rmIs348[F25B3.3p::hTDP-43::yfp]*	Morimoto lab
CMV0001	* rmIs348[F25B3.3p::hTDP-43::yfp]; daf-2 ( e1370 ) *	Voisine lab
